# Supplementary material for: Fasting alleviates metabolic alterations in mice with propionyl-CoA carboxylase deficiency due to Pcca mutation
Source: Commun Biol. 2024 May 29;7:659. doi: 10.1038/s42003-024-06362-8 (PMC11137003; doi:10.1038/s42003-024-06362-8)
Supplement: Supplementary file 2 — Supplementary Information [file 42003_2024_6362_MOESM2_ESM.pdf]

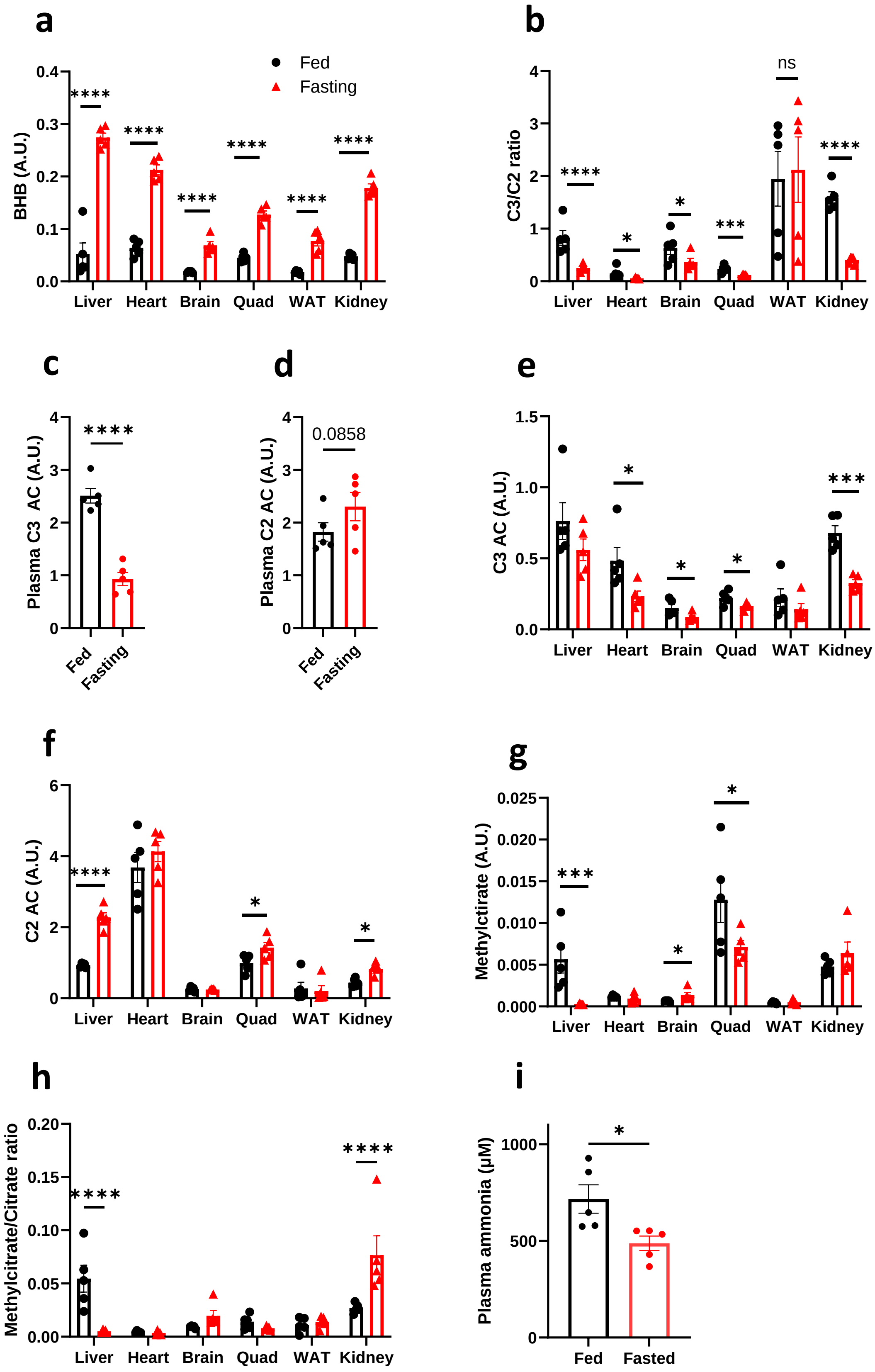
a
Fed
Fasting
BHB (A.U.)
0.0
0.1
0.2
0.3
0.4
****
Liver
Heart
Brain
Quad
WAT
Kidney
b
C3/C2 ratio
ns
****
*
***
0
1
2
3
4
c
Plasma C3 AC (A.U.)
Fed
Fasting
d
Plasma C2 AC (A.U.)
0.0858
e
C3 AC (A.U.)
0.0
0.5
1.0
1.5
f
C2 AC (A.U.)
0
2
4
6
g
Methylcitrate (A.U.)
0.000
0.005
0.010
0.015
0.020
0.025
h
Methylcitrate/Citrate ratio
0.00
0.05
0.10
0.15
0.20
i
Plasma ammonia (μM)
0
500
1000
Fed
Fasted

Supplemental Fig. 1. Impact of fasting on metabolism in *Pcca*$^{-/-}$(A138T) mice.

a,b Levels of 3-hydroxybutyrate (BHB) and ratio of C3 AC (propionylcarnitine) to C2 AC (acetylcarnitine) in tissues from fed and fasted (23-hour fasting) Pcca$^{-/-}$ (A138T) mice. **c,d** C3 AC and C2 AC levels in Plasma. **e-h** C3 AC levels, C2 AC levels, methylcitrate levels, and methylcitrate to citrate ratio in tissues from fed and fasted (23-hour fasting) *Pcca*$^{-/-}$ (A138T) mice. **i** Ammonia levels in plasma. Quad: quadriceps; WAT: white adipose tissue. N=5 per group. The error bar represents the SE. *, **, ***, **** indicate p-values less than 0.05, 0.01, 0.005, and 0.001, respectively.

a

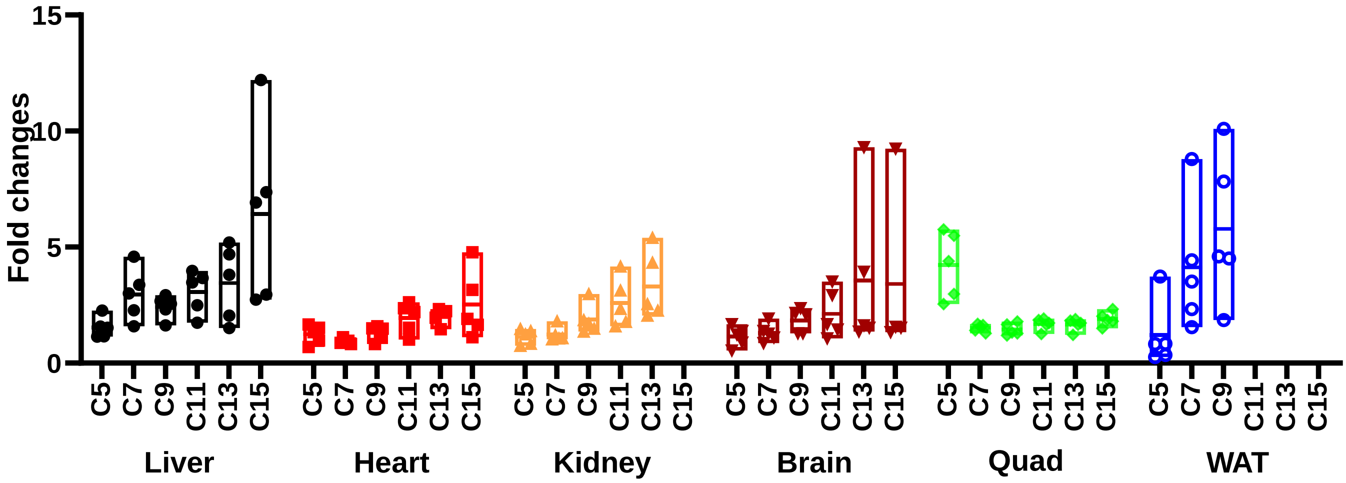

15
10
5
0
Fold changes
C5
C7
C9
C11
C13
C15
Liver
Heart
Kidney
Brain
Quad
WAT

b

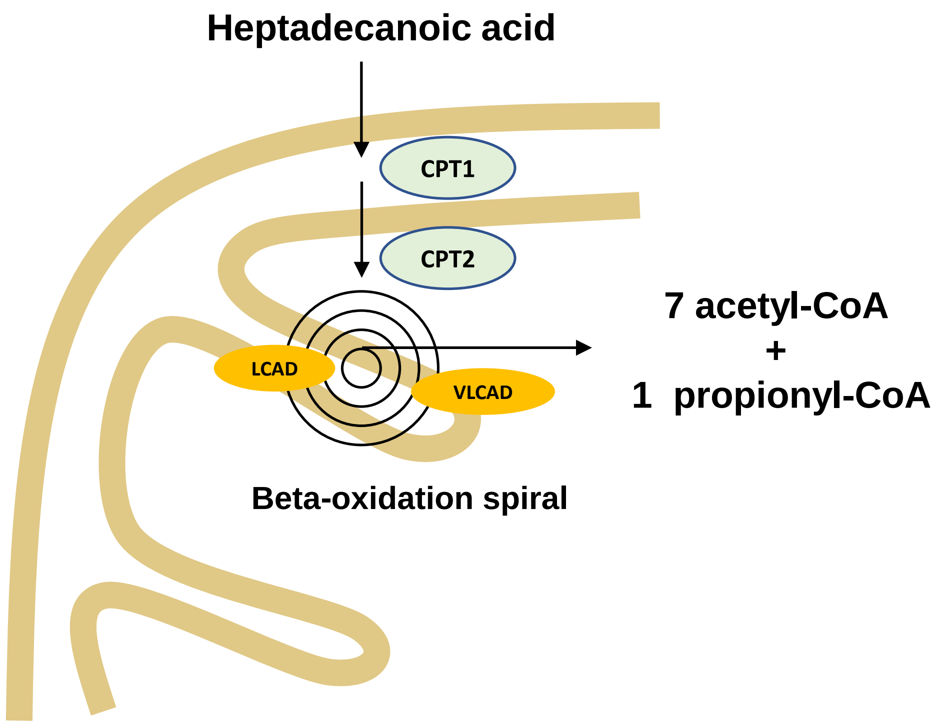

Heptadecanoic acid
CPT1
CPT2
LCAD
VLCAD
7 acetyl-CoA
+
1 propionyl-CoA
Beta-oxidation spiral

Supplemental Fig. 2. Odd-chain acylcarnitine changes in tissues of fasting *Pcca*$^{-/-}$ (A138T) mice.

a The fold changes in odd-chain acylcarnitines from C3 to C15 in the organs of *Pcca*$^{-/-}$ (A138T) mice after a 23-hour fasting vs the ones in the organs of fed *Pcca*$^{-/-}$(A138T) mice. Quad: quadriceps; WAT: white adipose tissue. **b** Schematic representation of heptadecanoic acid metabolism to acetyl-CoA and propionyl-CoA via complete beta-oxidation. CPT1: carnitine palmitoyltransferase 1, CPT2: carnitine palmitoyltransferase 2, LCAD: Long-chain acyl-CoA dehydrogenase, VLCAD: very long-chain acyl-CoA dehydrogenase. N=5 per group.

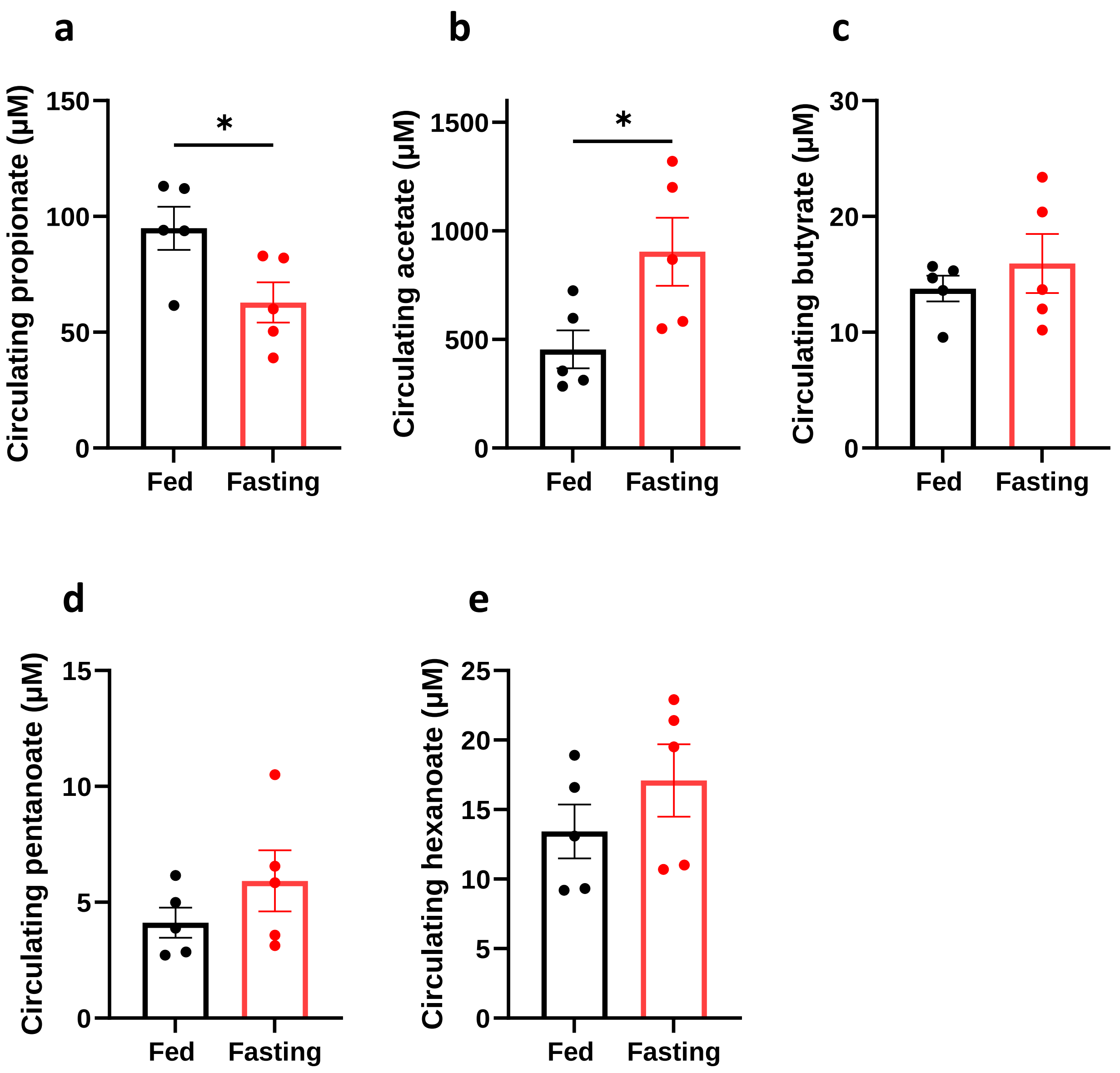

Supplemental Fig. 3. The circulating SCFAs levels in fed and fasted *Pcca*$^{-/-}$(A138T) mice.

To measure circulating SCFAs, blood samples were drawn from the inferior vena cava before mice were sacrificed. **a-e** Levels of propionate, acetate, butyrate, pentanoate, and hexanoate in circulating plasma from both fed and fasted (23-hour fasting) mice. N=5 per group. The error bar represents the SE. * indicates p-value less than 0.01.

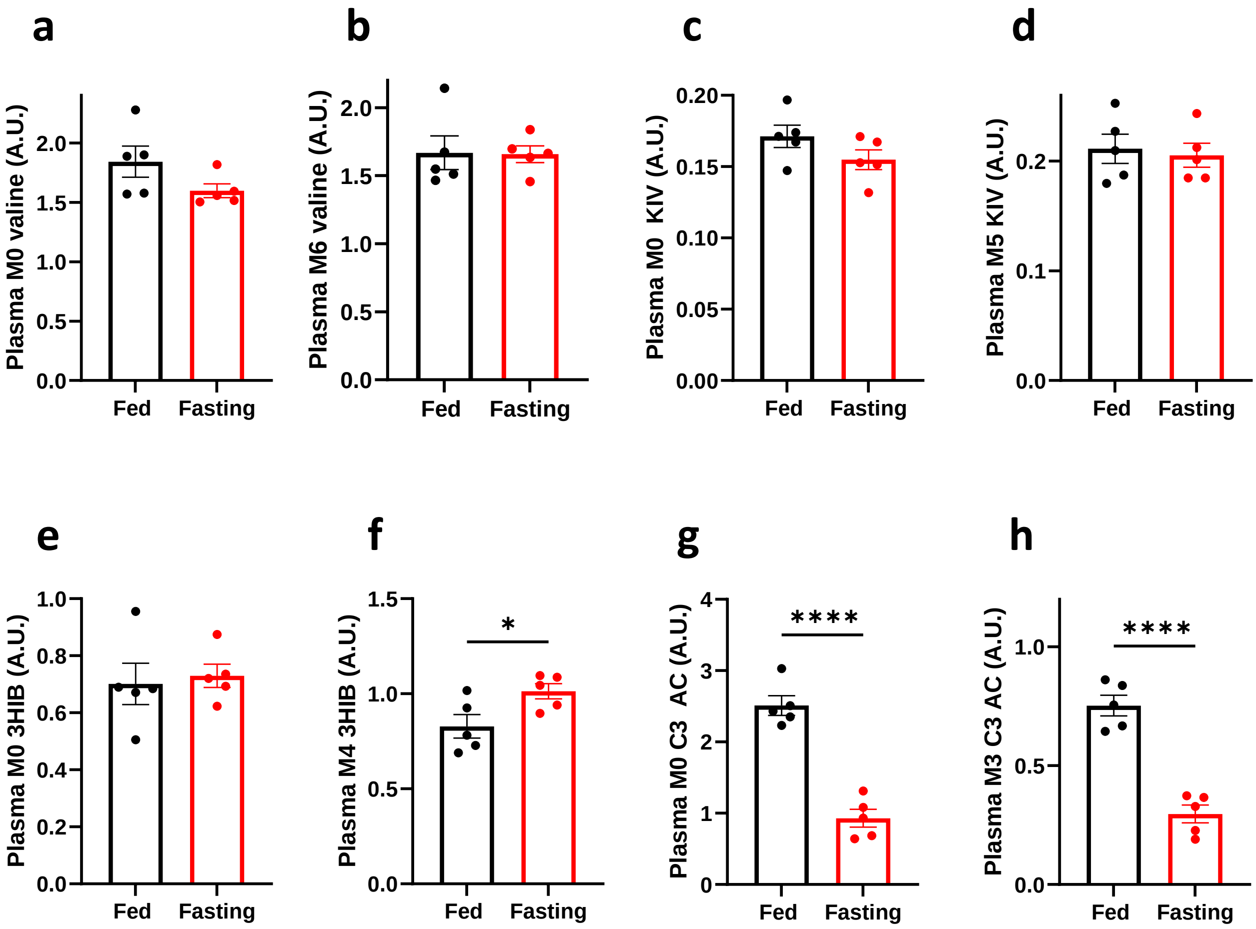

Supplemental Fig. 4. The plasma levels of metabolites of M6 valine in fed and fasted *Pcca*$^{-/-}$(A138T) mice.

a-h are plasma levels of M0 valine, M6 valine, M0 2-ketoisovalerate (M0 KIV), M5 2-ketoisovalerate (M5 KIV), M0 3-hydroxyisobutrate (M0 3HIB), M4 3-hydroxyisobutrate (M4 3HIB), M0 propionylcarnitine (M0 C3 AC), and M3 propionylcarnitine (M3 C3 AC) in the plasma of both fed and fasted (23-hour fasting) *Pcca*$^{-/-}$(A138T) mice. M0, M3, M5, and M6 denote the presence of 0, 3, 4, 5, and 6 heavy atoms, respectively, within a molecule. N=5 per group. The error bar represents the SE. * and **** indicate p-values less than 0.05 and 0.001, respectively.

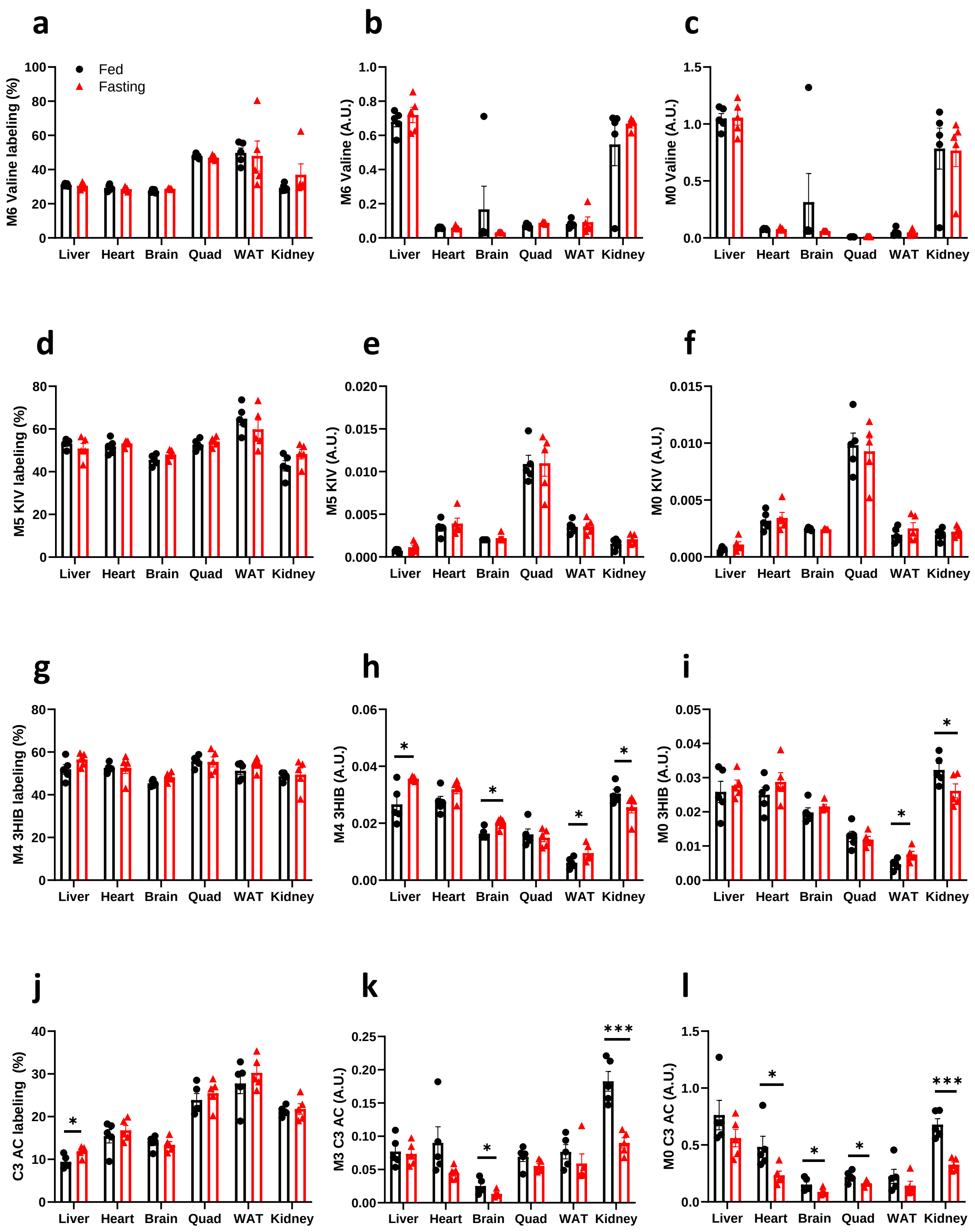

a
b
c
d
e
f
g
h
i
j
k
l
Fed
Fasting
M6 Valine labeling (%)
M6 Valine (A.U.)
M0 Valine (A.U.)
M5 KIV labeling (%)
M5 KIV (A.U.)
M0 KIV (A.U.)
M4 3HIB labeling (%)
M4 3HIB (A.U.)
M0 3HIB (A.U.)
C3 AC labeling (%)
M3 C3 AC (A.U.)
M0 C3 AC (A.U.)
Liver Heart Brain Quad WAT Kidney

Supplemental Fig. 5. Valine catabolism to propionyl-CoA remains unaltered in organs during fasting.

a-l Measurements of stable isotope labeling, including unlabeled/labeled levels of M0/M6 valine, M0/M5 2-ketoisovalerate (KIV), M0/M4 3-hydroxyisobutrate (3HIB), and M0/M3 propionylcarnitine (C3 AC) in the liver, heart, brain, quad, WAT, and kidney of both fed and fasted (23-hour fasting) *Pcca*$^{-/-}$(A138T) mice. M0, M3, M5, and M6 denote the presence of 0, 3, 4, 5, and 6 heavy atoms, respectively, within a molecule. Quad: quadriceps; WAT: white adipose tissue. N=5 per group. The error bar represents the SE. * and *** indicate p-values less than 0.05, 0.01, 0.005, and 0.001, respectively.

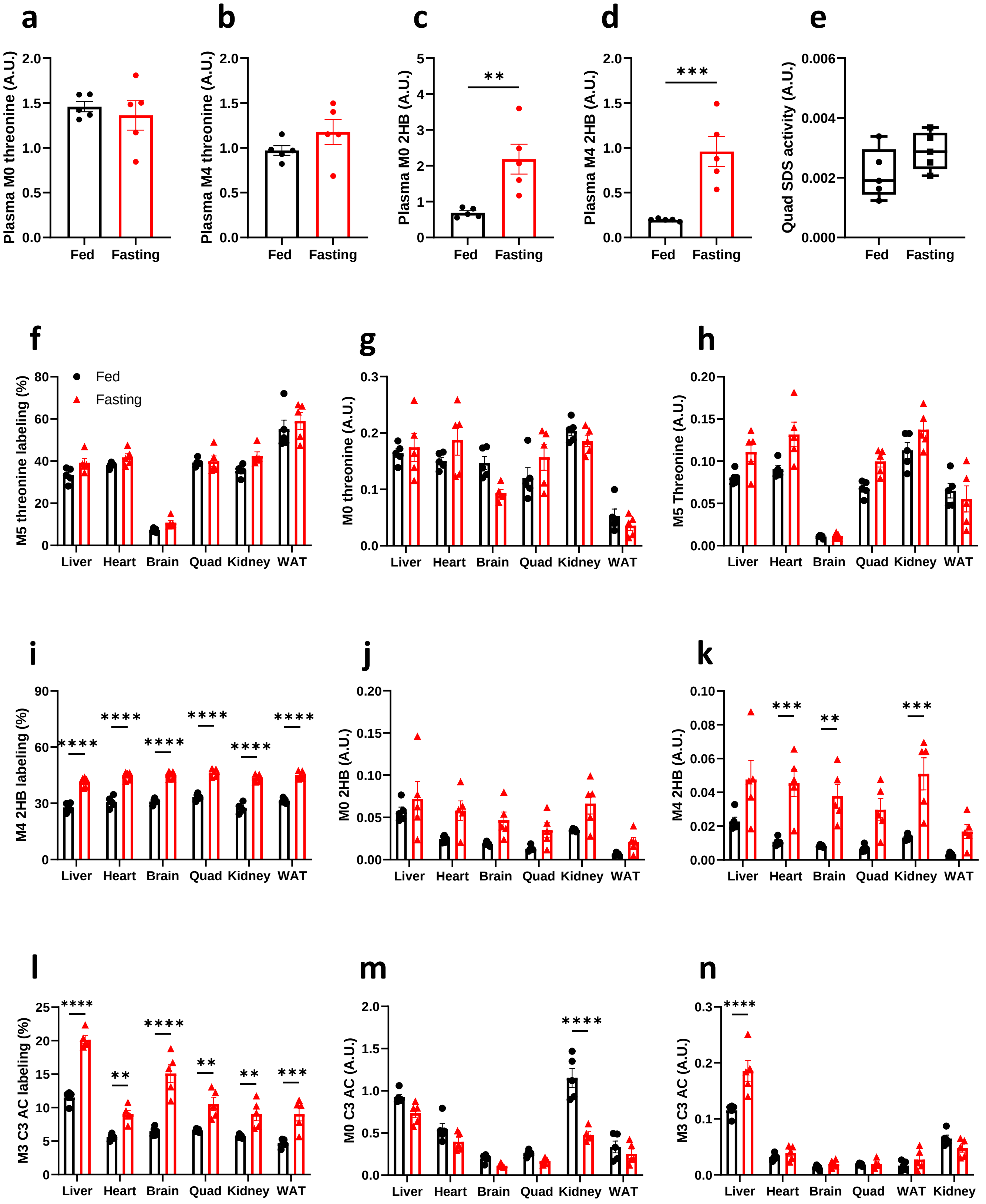
a
b
c
d
e
Plasma M0 threonine (A.U.)
Plasma M4 threonine (A.U.)
Plasma M0 2HB (A.U.)
Plasma M4 2HB (A.U.)
Quad SDS activity (A.U.)
Fed
Fasting
**
***
f
g
h
Fed
Fasting
M5 threonine labeling (%)
M0 threonine (A.U.)
M5 Threonine (A.U.)
Liver
Heart
Brain
Quad
Kidney
WAT
i
j
k
M4 2HB labeling (%)
M0 2HB (A.U.)
M4 2HB (A.U.)
****
***
**
l
m
n
M3 C3 AC labeling (%)
M0 C3 AC (A.U.)
M3 C3 AC (A.U.)

Supplemental Fig. 6. Increased threonine metabolism in fasted *Pcca*$^{-/-}$(A138T) mice.

a-d Plasma levels of M0 threonine, M4 threoninie, M0 2HB (2-hydroxybutyrate), and M4 2HB in plasma from both fed and fasted (23-hour fasting) *Pcca*$^{-/-}$(A138T) mice. **e** SDS activity in Quad of fed and fasted (23-hour fasting) *Pcca*$^{-/-}$(A138T) mice. SDS: Serine threonine dehydratase. **f-n** Isotope labeling and the levels of labeled/unlabeled M4/M0 threonine, M4/M0 2HB, and M3/M0 propionylcarnitine (C3 AC), in liver, heart, quad, kidney, and WAT from both fed and fasted (23-hour fasting) *Pcca*$^{-/-}$(A138T) mice. M0, M3, and M4 denote the presence of 0, 3, and 4 heavy atoms, respectively, within a molecule. Quad: quadriceps; WAT: white adipose tissue. N=5 per group. The error bar represents the SE. *, **, ***, and **** indicate p-values less than 0.05, 0.01, 0.005, and 0.001, respectively.

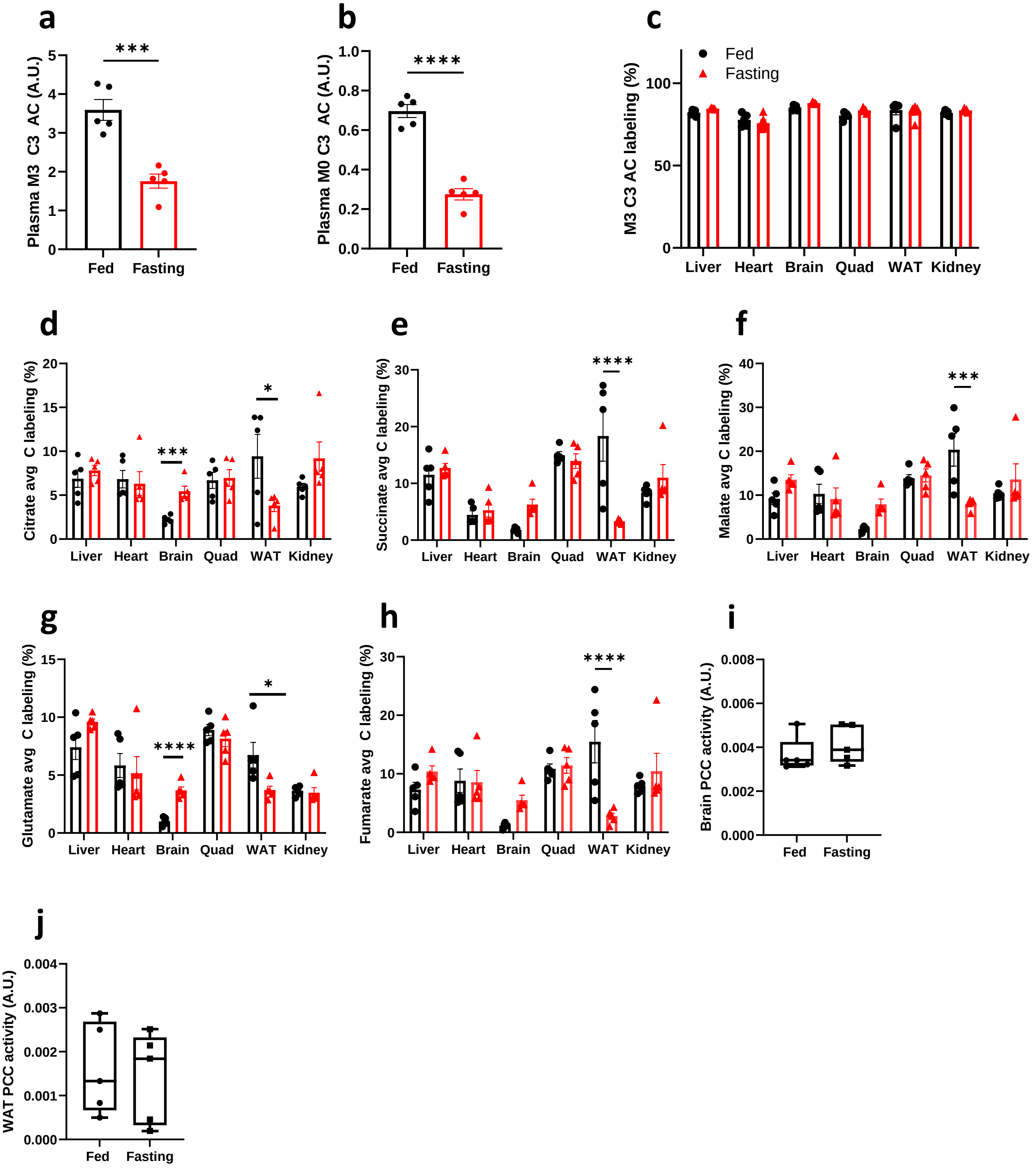
a
Plasma M3 C3 AC (A.U.)
***
Fed
Fasting
b
Plasma M0 C3 AC (A.U.)
****
Fed
Fasting
c
M3 C3 AC labeling (%)
Fed
Fasting
Liver
Heart
Brain
Quad
WAT
Kidney
d
Citrate avg C labeling (%)
***
*
Liver
Heart
Brain
Quad
WAT
Kidney
e
Succinate avg C labeling (%)
****
Liver
Heart
Brain
Quad
WAT
Kidney
f
Malate avg C labeling (%)
***
Liver
Heart
Brain
Quad
WAT
Kidney
g
Glutamate avg C labeling (%)
****
*
Liver
Heart
Brain
Quad
WAT
Kidney
h
Fumarate avg C labeling (%)
****
Liver
Heart
Brain
Quad
WAT
Kidney
i
Brain PCC activity (A.U.)
Fed
Fasting
j
WAT PCC activity (A.U.)
Fed
Fasting

Supplemental Fig. 7. The labeled metabolites derived from [$^{13}C_3$]propionate in fasted *Pcca*$^{-/-}$(A138T) mice.

a-c Plasma levels of M3 and M0 propionylcarnitine (C3 AC) and tissue labeling of M3 C3 AC from both fed and fasted (23-hour fasting) *Pcca*$^{-/-}$(A138T) mice. M0 and M3 denote the presence of 0 and 3 heavy atoms, respectively, within a molecule. **d-h** Average carbon labeling (avg C labeling) of citrate, succinate, malate, glutamate, and fumarate in liver, heart, brain, Quad, WAT, and kidney resulting from [$^{13}C_3$]propionate in both fed and fasted (23-hour fasting) *Pcca*$^{-/-}$(A138T) mice. Quad: quadriceps; WAT: white adipose tissue. **i-j** Measurement of brain and WAT PCC (propionyl-CoA carboxylase) activities in both fed and fasted (23-hour fasting) *Pcca*$^{-/-}$(A138T) mice. N=5 per group. The error bar represents the SE. *, **, ***, and **** indicate p-values less than 0.05, 0.01, 0.005, and 0.001, respectively.

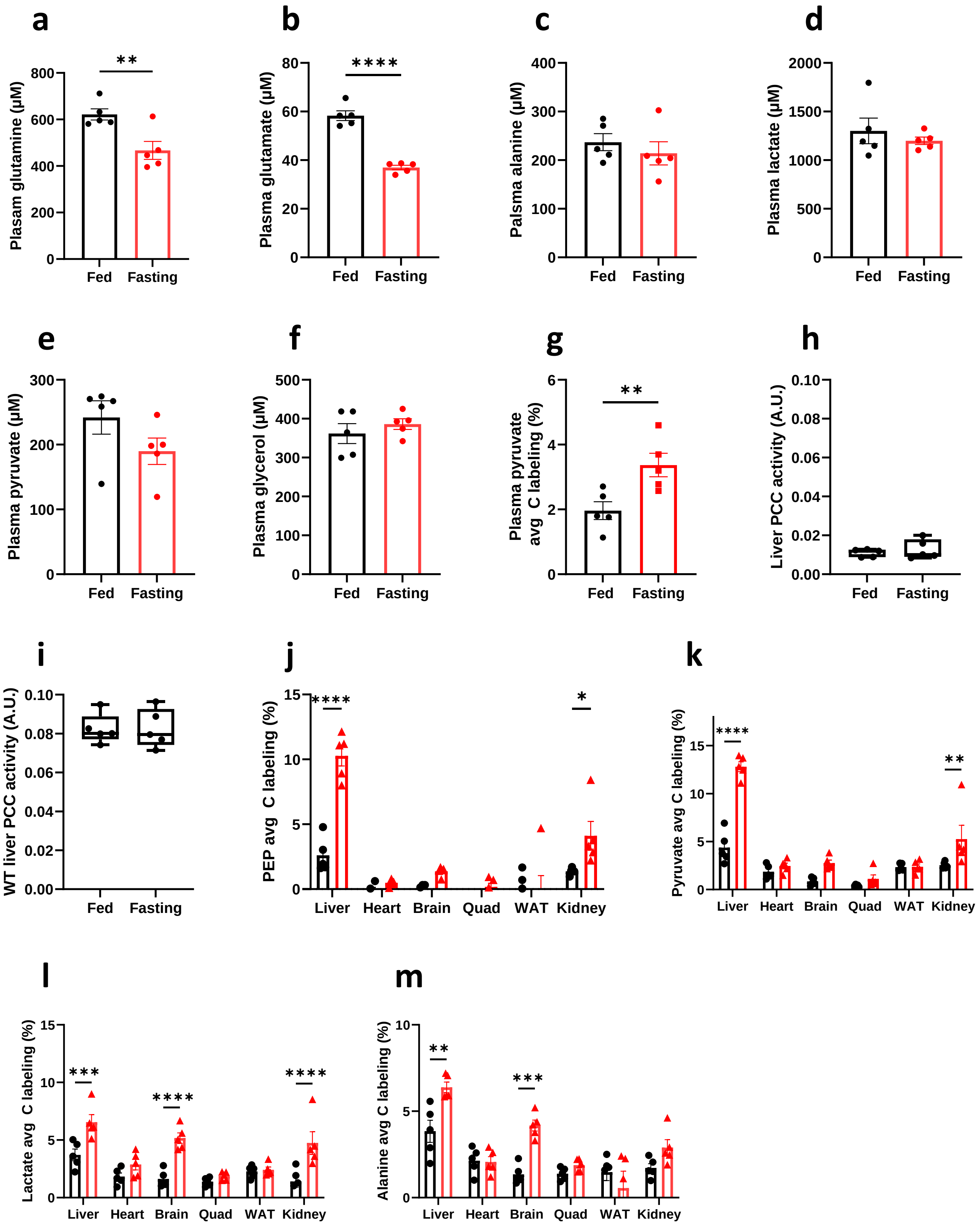
a
b
c
d
Plasam glutamine (μM)
Plasma glutamate (μM)
Palsma alanine (μM)
Plasma lactate (μM)
**
****
Fed
Fasting
e
f
g
h
Plasma pyruvate (μM)
Plasma glycerol (μM)
Plasma pyruvate avg C labeling (%)
Liver PCC activity (A.U.)
**
i
j
k
WT liver PCC activity (A.U.)
PEP avg C labeling (%)
Pyruvate avg C labeling (%)
****
*
**
Liver
Heart
Brain
Quad
WAT
Kidney
l
m
Lactate avg C labeling (%)
Alanine avg C labeling (%)
***
****
**
***

Supplemental Fig. 8. Increased gluconeogenesis in liver and kidney augments propionyl-CoA utilization in fasted *Pcca*$^{-/-}$(A138T) mice.

a-f Levels of circulating gluconeogenesis substrates, including glutamine, glutamate, alanine, lactate, pyruvate, and glycerol. **g** Average carbon labeling (avg C labeling) of pyruvate in the plasma from both fed and fasted (23-hour fasting) *Pcca*$^{-/-}$(A138T) mice. **h-i** Liver PCC activity in both fed and fasted (23-hour fasting) mice of *Pcca*$^{-/-}$(A138T) and wild type (WT). **j-m** Average carbon labeling (avg C labeling) of phosphoenolpyruvate (PEP), pyruvate, lactate, and alanine in the liver, heart, brain, quad, WAT, and kidney resulting from [$^{13}C_3$]propionate in both fed and fasted (23-hour fasting) *Pcca*$^{-/-}$(A138T) mice. Quad: quadriceps; WAT: white adipose tissue. N=5 per group. The error bar represents the SE. *, **, ***, and **** indicate p-values less than 0.05, 0.01, 0.005, and 0.001, respectively.
